# Development of simple sequence repeat (SSR) markers from a genome survey of Chinese bayberry (*Myrica rubra*)

**DOI:** 10.1186/1471-2164-13-201

**Published:** 2012-05-23

**Authors:** Yun Jiao, Hui-min Jia, Xiong-wei Li, Ming-liang Chai, Hui-juan Jia, Zhe Chen, Guo-yun Wang, Chun-yan Chai, Eric van de Weg, Zhong-shan Gao

**Affiliations:** 1 Department of Horticulture, The State Agriculture Ministry Laboratory of Horticultural Plant Growth, Development and Quality Improvement, Zhejiang University, Hangzhou 310058, China; 2 BGI-Shenzhen, Beishan Industrial Zone, Yantian District, Shenzhen, 518083, China; 3 Fruit Research Institute, Yuyao, Ningbo, 315400, China; 4 Forestry Technology Extension Center, Cixi Ningbo, 315300, China; 5 Plant Breeding-Wageningen University and Research Centre, P.O. Box 16, 6700 AA, Wageningen, The Netherlands

## Abstract

**Background:**

Chinese bayberry (*Myrica rubra* Sieb. and Zucc.) is a subtropical evergreen tree originating in China. It has been cultivated in southern China for several thousand years, and annual production has reached 1.1 million tons. The taste and high level of health promoting characters identified in the fruit in recent years has stimulated its extension in China and introduction to Australia. A limited number of co-dominant markers have been developed and applied in genetic diversity and identity studies. Here we report, for the first time, a survey of whole genome shotgun data to develop a large number of simple sequence repeat (SSR) markers to analyse the genetic diversity of the common cultivated Chinese bayberry and the relationship with three other *Myrica* species.

**Results:**

The whole genome shotgun survey of Chinese bayberry produced 9.01Gb of sequence data, about 26x coverage of the estimated genome size of 323 Mb. The genome sequences were highly heterozygous, but with little duplication. From the initial assembled scaffold covering 255 Mb sequence data, 28,602 SSRs (≥5 repeats) were identified. Dinucleotide was the most common repeat motif with a frequency of 84.73%, followed by 13.78% trinucleotide, 1.34% tetranucleotide, 0.12% pentanucleotide and 0.04% hexanucleotide. From 600 primer pairs, 186 polymorphic SSRs were developed. Of these, 158 were used to screen 29 Chinese bayberry accessions and three other *Myrica* species: 91.14%, 89.87% and 46.84% SSRs could be used in *Myrica adenophora*, *Myrica nana* and *Myrica cerifera,* respectively. The UPGMA dendrogram tree showed that cultivated *Myrica rubra* is closely related to *Myrica adenophora* and *Myrica nana*, originating in southwest China, and very distantly related to *Myrica cerifera*, originating in America. These markers can be used in the construction of a linkage map and for genetic diversity studies in *Myrica* species.

**Conclusion:**

*Myrica rubra* has a small genome of about 323 Mb with a high level of heterozygosity. A large number of SSRs were identified, and 158 polymorphic SSR markers developed, 91% of which can be transferred to other *Myrica* species.

## **Background**

Chinese bayberry is an important commercial horticultural crop. It has been cultivated for more than 7,000 years in southern China, but is little known elsewhere. The production area is currently 340,000 ha, with an annual production of 1.1 million tons. The plant is diploid (2n = 16), generally dioecious, with the female plants cultivated for fruit [1], growing well on poor soils due to the association of nitrogen-fixing bacteria with the root system. It is rich in anthocyanins exhibiting a wide range of pharmacological properties, such as anti-inflammatory, antitumor and antioxidative effects [2].

There are four species within the genus *Myrica* in China, namely *Myrica rubra* Sieb. & Zucc., *M*. *esculenta* Buch.-Ham., *M. nana* Cheval., and *M*. *adenophora* Hance. *M*. *rubra* is widely distributed, with many local cultivars in the Zhejiang, Jiangsu, Fujian and Guangdong provinces and a few from Guizhou, Yunnan and Hunan provinces. The best known cultivars are Biqi and Dongkui, both from the Zhejiang province. Although there are abundant germplasm resources, studies on genetics and breeding of the species are still in their infancy. Molecular marker technology is a popular tool for breeding and genetic research, and with the construction of a genomic library, 13 polymorphic microsatellite loci have been developed in *M. rubra*[3] and 11 from an expressed sequence tag library [4]. Recently, 12 primer pairs have been temporarily developed by ISSR-suppression PCR [5] with GSG (GT)_6_ as the primer for enriching microsatellite sequences. Reports on the genetic diversity in Chinese bayberry using SSR markers have also recently been published [6,7], but the number of markers for Chinese bayberry is limited.

The reproducibility, multiallelicism, co-dominance, relative abundance and good genome coverage of SSR markers have made them one of the most useful tools for genetic diversity and linkage mapping. Genomic SSRs and EST-SSRs, considered complementary to plant genome mapping, have been reported in many fruit crops, such as walnut [8], cherry [9], apricot [10] and coconut [11]. EST-SSRs are useful for genetic analysis, but their relatively low polymorphism and the high possibility of no gene-rich regions in the genome are limitations to their use. In contrast, genomic SSRs are highly polymorphic and tend to be widely distributed throughout the genome, resulting in better map coverage [12].

With genetic maps serving as the basis for future positional gene cloning, making map-based cloning and marker-assisted selection possible, the development of more SSRs is essential. As sequencing technologies advance, whole-genome shotgun (WGS) sequences are becoming increasingly available. These DNA sequences are valuable resources for SSR development in many plant species, such as rice [13] and papaya [14]. In addition, they can be used to evaluate the frequency and distribution of different types of SSRs in the genome, and even help to estimate genome size and characters such as heterozygosis and repeats.

As a way of reducing the cost of genotyping research, Schuelke [15] proposed a method for fluorescent dye labelling of PCR fragments with a sequence-specific forward primer: the universal fluorescent-labelled M13(-21) primer, at the 5^’^ end, acts as the forward primer in a ‘one-tube’ reaction. As this method allows for high-throughput genetic analyses, with a high number of microsatellite markers widely used, we considered the possibility of using this approach for multiplex PCR, to improve the efficiency and save costs.

In this study, we mined and validated 158 SSR markers and describe their application for understanding the genetic relationship among 29 Chinese bayberry accessions and other *Myrica* species. These markers are useful for genotyping and genetic diversity analysis and linkage mapping of *Myrica rubra* and other *Myrica* species.

## **Results**

### **Genome survey using whole genome shotgun data in Chinese bayberry**

**Figure 1:**
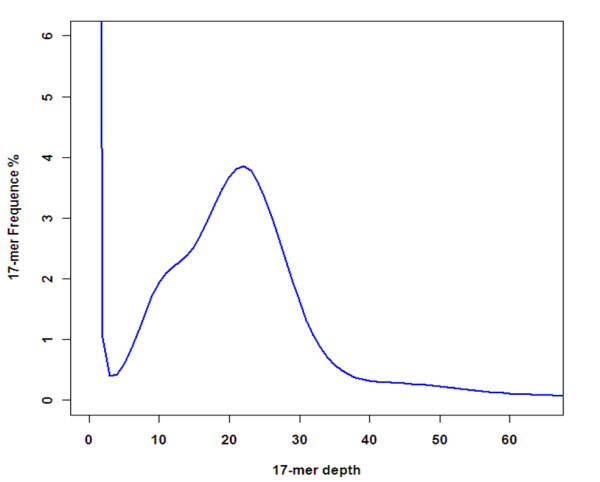
The distribution of 17-mer depth analysis based on whole genome shotgun data in Chinese bayberry.

WGS generated 273,161 (>100 bp) high quality sequence reads from two DNA libraries (250 bp and 500 bp) of the androphyte individual ‘C2010-55’. We used 9.01 G raw data for K-mer analysis and heterozygous simulation. For the 17-mer frequency distribution (Figure 1), the peak of the depth distribution was about 22. The estimated genome size was 323 Mb, using the formula: genome size = k-mer count/peak of the kmer distribution. The minor peak at 1/2 altitude of the main peak indicates the high level of heterozygosity in this genome (Figure 1). A total of 739,969 contigs were assembled with a total sequence length of 255.7 Mb. The length of N50 was 295 bp in our assembly, and the longest contig and scaffold 7,593 and 127,008 bp, respectively.

### **Frequency distribution of different types of SSR markers**

A total of 17,172 out of 273,161 scaffolds (6%) retrieved from the genome survey sequence contained 28,602 SSRs (Table 1), of which 5,401 contained more than one SSR, and 1,444 SSRs were present in compound format. Among the derived SSR repeats, the di-nucleotide was the most abundant repeat, accounting for 84.72% of total SSRs, followed by tri- (13.78%), tetra- (1.34%), penta- (0.12%), and hexa- (0.04%) nucleotides (Table 1). There was a large proportion of both dinucleotides and trinucleotides while the rest amounted to less 2%. The average frequency of occurrence was about 10.47% (Table 1).

**Table 1:** Occurrence of SSRs in the Genome Survey of Chinese bayberry

**Type**	**Number**	**Proportion in all SSRs (%)**	**Frequency (%)**
Dinucleotide	24,233	84.72%	8.87%
Trinucleotide	3,941	13.78%	1.44%
Tetranucleotide	383	1.34%	0.14%
Pentanucleotide	35	0.12%	0.013%
Hexanucleotide	10	0.04%	0.004%
Total	28,602	100%	10.47%

**Figure 2:**
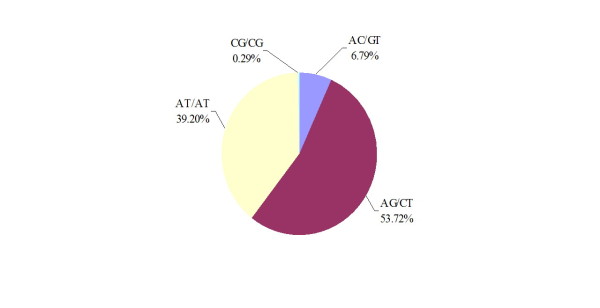
Percentage of different motifs in dinucleotide repeats in Chinese bayberry genome.

**Figure 3:**
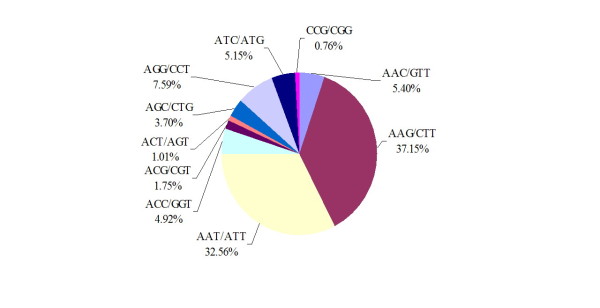
Percentage of different motifs in trinucleotide repeats in Chinese bayberry genome.

The SSR frequency of each motif is presented in Additional file 1. The SSR motif consists of 69 types. Among the repeat motifs of the dinucleotide, the AG/CT repeat was the most common, representing 53.72%, followed by 39.20% AT repeats (Figure 2), and the predominant motifs of trinucleotide (AAG/CTT and AAT/ATT) repeats accounted for 37.15% and 32.56%, respectively (Figure 3).

### **Polymorphism of SSR markers**

We first designed and synthesised 600 SSR primer pairs from those scaffolds more than 2Kb long. The majority of SSR loci were dinucleotide repeats (597, 99.5%), and the remainder trinucleotide. Initially, the effectiveness of these primer pairs was detected in two cultivars (Biqi and Dongkui) and *M. cerifera* through denaturing PAGE (Polyacrylamide gel electrophoresis), and 581 (96.8%) of these were amplified successfully in Biqi and Dongkui, and 400 (66.7%) in *M. cerifera*. The SSR loci (186, 31%) were identified as heterozygous loci either in Biqi or in Dongkui. Subsequently, they were used to screen 32 accessions, and detected an average of 8.25 alleles and from 3 to 15 alleles per locus (Table 2).

**Table 2:** Characteristics of 158 SSR markers in this study

**Locus**	**GenBank**	**Repeat motif**	**Primer sequence (5**^**'**^**-3**^**'**^**)**	**Size range(bp)**^**a**^	***Na***	***Ho***	***He***	**PIC**	***P***_**HW**_
	**Accession**								
ZJU001^ab^	JQ318696	(GA)10	F:<NED > <Tail-1 > CCTCTCCACCCATGAGAAAC	160-188	7	0.1667	0.4271	0.4002	0.0000
			R:CAAATCATTCCCTGCTTTCC						
ZJU002^ac^	JQ318697	(TC)13	F:<NED > <Tail-1 > TCAAAGAGACGTTGTGGCAG	219-229	4	0.2083	0.5257	0.4572	0.0005
			R:TCCGCTCACAGACAGAGAGA						
ZJU003^ab^	JQ318698	(AG)11	F:<NED > <Tail-1 > GTCACCTTGCTCTTCTTGGC	203-217	8	0.7407	0.8344	0.7949	0.0003
			R:TCCTTGTACTTGTTCTGCTGGA						
ZJU004^ac^	JQ318699	(GA)10	F:<NED > <Tail-1 > AACAGAACCATCGTCAAGGC	204-210	4	0.3571	0.7325	0.6704	0.0003
			R:GGTACAGTCGCTCCGGTTTA						
ZJU005^ab^	JQ318700	(AG)14	F:<NED > <Tail-1 > CTTTGGACATGGCAACACAC	200-228	11	0.3000	0.8679	0.8291	0.0000
			R:TCCACTTTGACAGATTCCCA						
ZJU006^ab^	JQ318701	(GA)10	F:<NED > <Tail-1 > CTCGCCCTCTCTCTCTACCA	193-205	5	0.2593	0.3305	0.3089	0.0000
			R:AGTTTATCCACCCGTGTCGT						
ZJU007^ab^	JQ318702	(AG)13	F:<NED > <Tail-1 > TGATCCATTGGAACCATGTG	193-209	8	0.5625	0.6617	0.6302	0.1868
			R:TCAGTTGATGGTGCAGAAGC						
ZJU008^ab^	JQ318703	(CT)10	F:<NED > <Tail-1 > GGAGAAATGAACGGTGGAGA	191-215	10	0.7931	0.7973	0.7563	0.0002
			R:TCCAAAGCTAATACCCACGC						
ZJU009^ab^	JQ318704	(CT)10	F:<NED > <Tail-1 > AATTGTCGCAAGTAGTCGCC	207-221	5	0.0741	0.3599	0.3371	0.0000
			R:ATATCAACCCATGGGAGCAA						
ZJU010^ab^	JQ318705	(CT)11	F:<NED > <Tail-1 > TGCAACATCGAAATTGGAAA	181-205	9	0.9032	0.8012	0.7614	0.0000
			R:ATGCCGGCAAGTCTTAGTGT						
ZJU011^a^	JQ318706	(GA)10	F:<NED > <Tail-1 > GGAGGCTCGTCAGTCATCTC	200-216	9	0.2692	0.7926	0.7554	0.0000
			R:TTAGCGTCCCTTCTCTCTCG						
ZJU012^ab^	JQ318707	(CT)12	F:<NED > <Tail-1 > CTTCACTCACCGCCTTTCTC	184-218	13	0.5000	0.8571	0.8251	0.0000
			R:AATGGCCTCCACATCTCAAG						
ZJU013^ab^	JQ318708	(CT)10	F:<NED > <Tail-1 > ACTTGTCATTCCCACGTTCC	211-221	6	0.4444	0.5199	0.4515	0.0094
			R:CACTCCATCTCAACCACCCT						
ZJU014^ab^	JQ318709	(AG)15	F:<NED > <Tail-1 > TGGAATGTCGATCTGAAACAA	186-212	13	0.6875	0.9033	0.8791	0.0251
			R:ACCAGCTTATACGACGGTGG						
ZJU015^ab^	JQ318710	(GA)11	F:<NED > <Tail-1 > TTGGTGTGGTGGTAATGGTG	199-221	6	0.6154	0.6614	0.5902	0.0585
			R:AAATAATGCAAGCAGGTGGG						
ZJU016^ab^	JQ318711	(TC)10	F:<NED > <Tail-1 > CCGTTGACTATTGCCCAGTT	196-216	11	0.6333	0.8469	0.8130	0.0179
			R:GGCAATTTCCAAATCGCTAA						
ZJU017^ab^	JQ318712	(CT)13	F:<NED > <Tail-1 > ACTGAAGAACCAAACGTGGG	180-200	6	0.6250	0.7093	0.6518	0.0003
			R:GGTGTGTTTCTCTGTGTGCG						
ZJU018^ab^	JQ318713	(CT)15	F:<NED > <Tail-1 > ACGAAATTTGACCAATCGCT	196-216	7	0.1429	0.7189	0.6667	0.0000
			R:AGGGTTTCTTCTGGTTCGGT						
ZJU019^ab^	JQ318714	(GA)12	F:<NED > <Tail-1 > TTTCATAACCCGTTGGCTTC	209-219	6	0.2800	0.6865	0.6317	0.0000
			R:AAGGTGGAAACGTGTCAAGG						
ZJU020^b^	JQ318715	(AG)10	F:<NED > <Tail-1 > CACAGGACATGTGATGGAGG	201-213	7	0.5172	0.7453	0.6983	0.0000
			R:CCATCCTGAGCTTTGTCGAT						
ZJU021^a^	JQ318716	(TG)10	F:<NED > <Tail-1 > TCGCCAGCTTCCTAATGTCT	190-212	8	0.7778	0.7428	0.7025	0.0663
			R:GAGCGCATGTTGTTGCTAAA						
ZJU022^ab^	JQ318717	(GA)10	F:<NED > <Tail-1 > AAGCTTAAGCAAGCGTCGAG	188-208	9	0.6923	0.8575	0.8227	0.0109
			R:TGCGAAGGGAAATTTCAGAC						
ZJU023^ac^	JQ318718	(AG)15	F:<NED > <Tail-1 > GTGTTTGGGCAGCACCTATT	200-226	14	0.6667	0.8840	0.8544	0.0251
			R:AAAGAGTACAACAACGCGGG						
ZJU024^ab^	JQ318719	(TC)10	F:<NED > <Tail-1 > CCGCATGTTTGATTGATGTC	180-196	6	0.6000	0.7345	0.6716	0.1624
			R:GCGTTGAGCGGAGAGATTAC						
ZJU025^ab^	JQ318720	(TC)10	F:<NED > <Tail-1 > TTTGAGCGATAGTACGGAGG	216-234	8	0.2667	0.7537	0.7044	0.0000
			R:ATATGCTACGTTGGTTGCCC						
ZJU026^ab^	JQ318721	(TC)10	F:<NED > <Tail-1 > CCAGACAGGTTAGCCACCAT	200-220	10	0.4545	0.8573	0.8199	0.0000
			R:GCCTCTGGATCTCGATTACG						
ZJU027	JQ318722	(TTC)8	F:<NED > <Tail-1 > GTTGCAATTTGCCTCCATTT	203-227	6	0.3125	0.6250	0.5321	0.0003
			R:GGTGCCTATACTGCCAGCTC						
ZJU028^ab^	JQ318723	(AG)10	F:<NED > <Tail-1 > CAACCATCCAAACCAAATCC	164-170	4	0.1724	0.2789	0.2566	0.0000
			R:TCTACCAATCGTGGCTAGGG						
ZJU029^ab^	JQ318724	(AG)10	F:<NED > <Tail-1 > TCTTCCGGGATGTCTACAGG	189-205	6	0.5312	0.6925	0.6296	0.0480
			R:CAACAGCAATCGCAAAGAAA						
ZJU030^ab^	JQ318725	(CA)13	F:<NED > <Tail-1 > AAGTGAGCTCTCCCTCCCTC	193-205	7	0.4286	0.7208	0.6676	0.0000
			R:CACCGAAATACTTGCCGTTT						
ZJU031^ab^	JQ318726	(GA)16	F:<NED > <Tail-1 > GCACAGGAACACCAGGATCT	179-195	8	0.8387	0.7948	0.7492	0.0000
			R:CCAAGCCCTAATTCCCTTTC						
ZJU032^ab^	JQ318727	(TC)11	F:<NED > <Tail-1 > ATTCCCACGTTCGTTCAGAC	204-226	8	0.6786	0.6442	0.5852	0.0220
			R:GATGCCTAACTCCGAATCCA						
ZJU033^ab^	JQ318728	(TC)10	F:<NED > <Tail-1 > GCACAAGTTGCTGACATGCT	195-207	6	0.0690	0.6655	0.5897	0.0000
			R:AGTTGCATTCAACCCACACA						
ZJU034^ab^	JQ318729	(CT)10	F:<NED > <Tail-1 > ATGGGAATGTGGAGAACGAG	191-209	8	0.4138	0.7762	0.7250	0.0000
			R:GCTTTGCTTCTTTGCTTTGG						
ZJU035^ab^	JQ318730	(GA)14	F:<NED > <Tail-1 > TTGGATCCTGGTTACCTTCG	201-217	8	0.1290	0.7425	0.6900	0.0000
			R:AAACTGCATGCATGGTTCCT						
ZJU036^ab^	JQ318731	(GA)10	F:<NED > <Tail-1 > CTGCCACTCTTACTGGCCTC	186-214	8	0.3333	0.5895	0.5516	0.0000
			R:ATGTGCCCAATCTTGACTCC						
ZJU037^ab^	JQ318732	(TC)10	F:<NED > <Tail-1 > GTGATTTCCCTCCCATTGAC	208-228	9	0.8125	0.7867	0.7429	0.0135
			R:ACGAAGCGGGAAGTAGGATT						
ZJU038^b^	JQ318733	(AG)10	F:<NED > <Tail-1 > CTTATGGCCCGTTTGTAACC	194-200	4	0.2273	0.5106	0.4646	0.0007
			R:AACGATTGCTTTAAGCGGAA						
ZJU039^a^	JQ318734	(CT)10	F:<NED > <Tail-1 > AAACGAAAGTGGGCGTATTG	219-229	6	0.3077	0.6161	0.5745	0.0004
			R:CACCAGTGCGTCCTATGAGA						
ZJU040	JQ318735	(TC)16	F:<NED > <Tail-1 > AAACTCCGTGCTGGAATCAA	198-220	10	0.3182	0.8192	0.7798	0.0000
			R:GCAGACAAGCCTTCCTGTTC						
ZJU041^ab^	JQ318736	(TC)11	F:<PET > <Tail-2 > TGATCACCTTTCAGTTGGCA	226-244	5	0.2258	0.3199	0.3031	0.0000
			R:CACATTGGCAGAGTCCTGAA						
ZJU042^ab^	JQ318737	(TC)10	F:<PET > <Tail-2 > AGGATTTCTCCAGAGGGACG	220-242	5	0.3571	0.5331	0.4880	0.0000
			R:GGTTCCGCATCAAACTACAAA						
ZJU043^b^	JQ318738	(CT)10	F:<PET > <Tail-2 > AAACCGAGCTCTCCTAAGCC	225-245	4	0.5714	0.6383	0.5667	0.2655
			R:CTCGCAATTTCTCGGGATAC						
ZJU044^ab^	JQ318739	(GA)12	F:<PET > <Tail-2 > GATGGTGGCTTGTCTTGGTT	235-255	8	0.2500	0.5091	0.4853	0.0000
			R:AAGTGGGACGTCAATTCCTG						
ZJU045^ab^	JQ318740	(CT)10	F:<PET > <Tail-2 > GAGAGAGGGAGAGAGGCCAT	228-258	13	0.6129	0.8821	0.8544	0.0007
			R:GGAAGATTCATGGGAGAGGG						
ZJU046^ab^	JQ318741	(AG)10	F:<PET > <Tail-2 > TTGCTGTAAGCATCGCAATC	226-242	7	0.3871	0.6256	0.5824	0.0000
			R:AAGCTCCGGTAACACACACC						
ZJU047^ab^	JQ318742	(GA)13	F:<PET > <Tail-2 > TTCGATCATTCATGAGGCTG	247-259	7	0.7097	0.7615	0.7074	0.0019
			R:TTAATTGCATCCCGGATTTC						
ZJU048^ab^	JQ318743	(CT)14	F:<PET > <Tail-2 > AGCGGACCGAGTTGTAGAGA	230-254	12	0.2903	0.8493	0.8166	0.0000
			R:CCAACCCTACAAAGCGAGAG						
ZJU049^ab^	JQ318744	(GAA)8	F:<PET > <Tail-2 > GTGTCTGCAGCAACTTCCAC	234-267	10	0.8125	0.7262	0.6797	0.0000
			R:GTCGGAACCGAAGATGGTTA						
ZJU050^ab^	JQ318745	(AG)11	F:<PET > <Tail-2 > GTCACAGCCTGGATAGCTCC	233-245	7	0.3000	0.7288	0.6916	0.0000
			R:GTCTCTCCTGGATGAGCTGC						
ZJU051^ab^	JQ318746	(AG)12	F:<PET > <Tail-2 > AGAGAAAGACCGGGACCAAT	229-233	3	0.4333	0.4198	0.3594	0.0012
			R:GAGAAATAAAGCCGAGCGTG						
ZJU052^ab^	JQ318747	(AG)16	F:<PET > <Tail-2 > CCCGAGCTGAACGAAATAGA	230-248	9	0.4348	0.8628	0.8261	0.0000
			R:GGATCAAAGCGTTGTCGTTT						
ZJU053^ab^	JQ318748	(AG)10	F:<PET > <Tail-2 > AAATCCGAAACACCTCTCCC	222-240	8	0.5000	0.5655	0.5211	0.0001
			R:ATGTGGAGACTTCCACTGGG						
ZJU054^ab^	JQ318749	(CT)13	F:<PET > <Tail-2 > TTGATTTGCTTTGTGCATTTG	232-250	9	0.3000	0.8667	0.8268	0.0003
			R:CAAACTACCGTGCCCAACAT						
ZJU055^ab^	JQ318750	(CT)10	F:<PET > <Tail-2 > TTATGGGTTTCATTGGGCAG	238-254	6	0.2500	0.7006	0.6357	0.0000
			R:TCACCAGGCTACTGCATGTC						
ZJU056^ab^	JQ318751	(GA)13	F:<PET > <Tail-2 > GACAAAGTGGGTGCCATTCT	230-246	7	0.5714	0.7643	0.7122	0.0068
			R:TGCATGCTTCCTTTCTTCCT						
ZJU057^ab^	JQ318752	(CT)10	F:<PET > <Tail-2 > TCATGTGGAGATTGAAGCCA	230-244	6	0.1579	0.6814	0.6283	0.0000
			R:CGTCCCGAATGAAGATTTGT						
ZJU058^ab^	JQ318753	(GT)10	F:<PET > <Tail-2 > TCCGGAGCTTTCAATCTCAT	252-274	11	0.7500	0.8274	0.7900	0.8036
			R:GCCTACGAACTCAGGTTCCA						
ZJU059^b^	JQ318754	(TC)14	F:<PET > <Tail-2 > TGTTTGTTTCTTGCTATTTCCATC	217-235	7	0.5200	0.7935	0.7505	0.0016
			R:GACAGTTCCCACCAGCATTT						
ZJU060^ab^	JQ318755	(GT)8(GA)9	F:<PET > <Tail-2 > TGGCCAGGAACTTTGTATCC	223-243	7	0.6562	0.8110	0.7691	0.0000
			R:GAAAGATTGGGAATGCTGGA						
ZJU061^ab^	JQ318756	(TC)11	F:<PET > <Tail-2 > TTTGGAGGAAGCAAACAAGC	204-232	11	0.2812	0.7922	0.7506	0.0000
			R:TCCTGCGCCAACAATCTAAT						
ZJU062	JQ318757	(TC)10	F:<PET > <Tail-2 > GTCGAGAGAACAAAGCGACC	240-252	7	0.2400	0.3282	0.3135	0.0004
			R:GTCCAATGCCGCACTAACTT						
ZJU063^ab^	JQ318758	(TC)12	F:<PET > <Tail-2 > ACTCAGCAGGACCACCAACT	232-250	10	0.7000	0.8593	0.8270	0.1320
			R:TTAGACGGAAATTGGGCTTG						
ZJU064^b^	JQ318759	(GA)10	F:<PET > <Tail-2 > ACCATGAAGCTGACCTGGAG	226-244	6	0.4348	0.7256	0.6666	0.0001
			R:TTTCGTGGTCCCACCTACTC						
ZJU065^ac^	JQ318760	(CA)13	F:<PET > <Tail-2 > TCCAGAATATCATCTCTTGCCA	214-236	9	0.6333	0.7706	0.7219	0.0001
			R: ATATTCCTAACGTGTGCGGG						
ZJU066^ab^	JQ318761	(GA)10	F:<PET > <Tail-2 > CTTTCCCTTGTCGCTTTCAG	221-235	8	0.2593	0.6450	0.6075	0.0000
			R:GGTCGCAGATCAGGTCAAGT						
ZJU067^ab^	JQ318762	(CT)10	F:<PET > <Tail-2 > CAGACAGCGAGGAGACAACA	217-263	11	0.6923	0.8273	0.7861	0.0070
			R:GGTCTTTCGAACTTTGCTCG						
ZJU068^ab^	JQ318763	(CT)10	F:<PET > <Tail-2 > GAAGCTAAACGCCAGAAACG	227-239	6	0.2917	0.7535	0.6913	0.0000
			R:ACTCCTCACACGAATGGGTC						
ZJU069^bc^	JQ318764	(GA)10	F:<PET > <Tail-2 > TGCCATAATCCTGAGAGCCT	224-258	8	0.2609	0.5594	0.5235	0.0004
			R:TGTTCTGTAATGGCGTGGAA						
ZJU070^ab^	JQ318765	(CT)11	F:<PET > <Tail-2 > GTGCTCGAGATGTCCTCCAT	221-247	7	0.5200	0.7861	0.7364	0.0000
			R:ACAATCCCATCGCATACAGG						
ZJU071^ab^	JQ318766	(GA)10	F:<PET > <Tail-2 > CTAAGGTTGGTCCCTGTCCA	228-234	3	0.3704	0.6157	0.5305	0.0110
			R:CTTGTGTGGTGATGGTTTGG						
ZJU072^ab^	JQ318767	(AG)10	F:<PET > <Tail-2 > AGTCAGCGTGGGAATGTACC	223-237	7	0.5625	0.7604	0.7117	0.0000
			R:TTTCAGAACAAGTTCGTCGC						
ZJU073^a^	JQ318768	(AG)12	F:<PET > <Tail-2 > TACGCCAAGATCCAAAGACC	222-242	7	0.2105	0.7568	0.7087	0.0000
			R:TCTCGAGTTGAGTTTGGGCT						
ZJU074^ab^	JQ318769	(CT)15	F:<PET > <Tail-2 > TGCAGAGGAACTGGTGACTG	215-239	10	0.5517	0.8234	0.7831	0.0007
			R:GAGAAGGCTCAGTGGGTGAG						
ZJU075^b^	JQ318770	(CT)17	F:<PET > <Tail-2 > AATAAACACACAGGGCGAGG	239-255	9	0.0769	0.8650	0.8307	0.0000
			R:ATCGGGCAGACCAGAATATG						
ZJU076^ab^	JQ318771	(AG)9	F:<FAM > <Tail-3 > ATGGTTACCGACGTCCTCTG	131-169	11	0.8438	0.8353	0.8034	0.0000
			R:AGTGCAGAGTGCGAGATCAA						
ZJU077^ab^	JQ318772	(AC)9	F:<FAM > <Tail-3 > TTTGGAATTCAACAACATTTAGAC	137-153	8	0.2000	0.6590	0.6079	0.0000
			R:TGCAGCCTTGCTCCTCTTAT						
ZJU078^ab^	JQ318773	(TC)10	F:<FAM > <Tail-3 > ACACCACGGTTCTTCGATTC	130-146	6	0.5500	0.7513	0.6881	0.1339
			R:GTAACGAGGCTCTTGCTTGC						
ZJU079^ab^	JQ318774	(TC)13	F:<FAM > <Tail-3 > AAGGCTAGACCGCAATCTGA	116-134	9	0.8438	0.8596	0.8291	0.0008
			R:GGGCAACAGTTTACTTCCCA						
ZJU080^ab^	JQ318775	(CT)9	F:<FAM > <Tail-3 > CTTGACGAAATGCAGACGAA	124-134	5	0.2903	0.3411	0.3172	0.0103
			R:TCCGGATCAGGGTCAAATAG						
ZJU081^ab^	JQ318776	(GA)8	F:<FAM > <Tail-3 > TGCTCTTGCAGAGAGTCGAG	137-157	6	0.5517	0.5820	0.5379	0.0003
			R:TCATAATACCCTTGGCAAACA						
ZJU082^ab^	JQ318777	(CT)10	F:<FAM > <Tail-3 > TATATCGAATCCCAAAGGCG	129-141	5	0.3438	0.4043	0.3792	0.0169
			R:AAGATATTGGTCCGGCTCCT						
ZJU083^ab^	JQ318778	(AG)10	F:<FAM > <Tail-3 > TAGCCTTGGAGATTTAGGGC	133-157	11	0.8667	0.8960	0.8692	0.0000
			R:TTGAAATTTCGCAGCCTCTT						
ZJU084^ab^	JQ318779	(AG)9	F:<FAM > <Tail-3 > TTTCGATTGGTGGTCTGTGA	124-138	6	0.1379	0.5197	0.4766	0.0000
			R:TTATTAACTTCACTTTGTTTATTCGG						
ZJU085^ab^	JQ318780	(AG)9	F:<FAM > <Tail-3 > GCTTTAACCGAGTGATGGGA	150-184	8	0.6875	0.5992	0.5383	0.6352
			R:TAAAGGAGCGCTGGAAAGAA						
ZJU086^ab^	JQ318781	(TC)10	F:<FAM > <Tail-3 > TCCTCTCTTTCACACTTCCGA	118-152	13	0.9062	0.8720	0.8445	0.0005
			R:GGTCGATCATTTCTCTCCCA						
ZJU087^ab^	JQ318782	(GA)9	F:<FAM > <Tail-3 > CGAGTGTAGCTAGGAACGGC	135-149	8	0.4688	0.7748	0.7273	0.0204
			R:AATTGGACCTGCAAATCTCG						
ZJU088^ab^	JQ318783	(CT)9	F:<FAM > <Tail-3 > GAGCTCCGAACTTCTTCCCT	126-150	13	0.9677	0.8773	0.8490	0.0053
			R:CTTCTCCACAGGACTCTGCC						
ZJU089^ab^	JQ318784	(GA)8	F:<FAM > <Tail-3 > CGTTAGGATTCGGGAACAGA	138-152	7	0.8065	0.7382	0.6778	0.0000
			R:CAGGGCTAATGTGGACCAGT						
ZJU090^ab^	JQ318785	(AG)9	F:<FAM > <Tail-3 > GGAAATCTCCGAATGTGATCC	118-134	8	0.2903	0.6642	0.6089	0.0000
			R:TGGTGGATGAACCACTCAAA						
ZJU091^bc^	JQ318786	(TC)15	F:<FAM > <Tail-3 > AAAGAGCACACAGCCCTAGC	124-146	10	0.4615	0.8695	0.8358	0.0012
			R:GGCAGTGTCGCAGTGATAGA						
ZJU092^ab^	JQ318787	(TG)10	F:<FAM > <Tail-3 > CTCTTGCCGACCTCATTGTT	127-151	11	0.6875	0.8264	0.7916	0.0041
			R:CGGGACTCGCATAAATCACT						
ZJU093^ab^	JQ318788	(GA)10	F:<FAM > <Tail-3 > ATGCCATGTTGCATGAGTGT	130-156	12	0.9355	0.8662	0.8367	0.3689
			R:TATCCCGTAAGCAATCAGGG						
ZJU094^ab^	JQ318789	(CT)10	F:<FAM > <Tail-3 > ATCACGGGTTCTGCTGTTCT	124-150	10	0.9062	0.8646	0.8332	0.0000
			R:CAGAAGAAGCCATTTCTGCC						
ZJU095^ab^	JQ318790	(AG)9	F:<FAM > <Tail-3 > TACCCACCGTACCAAAGGTC	114-130	7	0.4839	0.7070	0.6420	0.0004
			R:GAATGAACCCAGGCGATAGA						
ZJU096^ab^	JQ318791	(CT)10	F:<FAM > <Tail-3 > CATACTGCAATGCATCTCCC	126-154	13	0.8000	0.8757	0.8479	0.0310
			R:TCAATTTGTGTGCCCTTACG						
ZJU097^ab^	JQ318792	(AG)10	F:<FAM > <Tail-3 > AATTGTTAGGGAGGGCTCGT	118-134	8	0.8438	0.7778	0.7297	0.0009
			R:TGCGTTGTGGAGACCATTTA						
ZJU098^ab^	JQ318793	(CT)9	F:<FAM > <Tail-3 > GACGCTCCATCTCTGGTCTC	145-167	10	0.9355	0.8831	0.8549	0.0483
			R:CCCAAACCGCACTAGAGAAA						
ZJU099^ab^	JQ318794	(GA)10	F:<FAM > <Tail-3 > TTGTTGCACTTGTGGGTGAT	130-150	9	0.7742	0.7763	0.7299	0.0000
			R:AACTACAAACAGCCCAACCG						
ZJU100^ab^	JQ318795	(TC)9	F:<FAM > <Tail-3 > ACTTGTCCGGATTCCACAAC	128-154	5	0.8333	0.6316	0.5629	0.2930
			R:TCAAGGCACACAATAATGCAA						
ZJU101^ab^	JQ318796	(AG)9	F:<FAM > <Tail-3 > TGATTGAGCTGCCAACAAAG	134-154	7	0.6667	0.7062	0.6527	0.8110
			R:TTTAACATTTGGCACCGACA						
ZJU102^ab^	JQ318797	(GA)10	F:<FAM > <Tail-3 > GAACCACGAACTTCAACCGT	118-132	8	0.4231	0.5890	0.5441	0.0111
			R:AACCACCAAACTTAGCTTCCA						
ZJU103^ab^	JQ318798	(AG)9	F:<FAM > <Tail-3 > TGAGGAGGGAGTTGAGTTGG	121-139	10	0.7097	0.7731	0.7359	0.0003
			R:GCGTCTTCCTCCTCCTTCTT						
ZJU104^ab^	JQ318799	(TA)9	F:<FAM > <Tail-3 > ACGTGGCAGTTGAGTTGTTG	114-128	6	0.3704	0.6296	0.5702	0.1383
			R:TCAGATCTCCGTTGGAGCTT						
ZJU105^ab^	JQ318800	(GA)11	F:<FAM > <Tail-3 > TGAGAAACGCAGCAAGAGAA	135-157	11	0.5806	0.8165	0.7801	0.0000
			R:CATCTCTCCCAAGCATCCTC						
ZJU106^ab^	JQ318801	(GA)8	F:<FAM > <Tail-3 > GCAGTCGGATAGAGAGACGG	134-146	7	0.3636	0.7717	0.7203	0.0000
			R:TGTTGATCAAACACACCGAGA						
ZJU107^ab^	JQ318802	(TC)10	F:<FAM > <Tail-3 > TGGTGTCACGATTCACTGGT	114-130	8	0.4375	0.5322	0.5012	0.3632
			R:CTGCATGTAATGGCAGTTCAA						
ZJU108^b^	JQ318803	(CT)9	F:<FAM > <Tail-3 > TTGGTAGTGCACTGCAGGAG	132-160	13	0.3929	0.8253	0.7909	0.0000
			R:CGAGGGTCGAGTTCAGAGAG						
ZJU109^ab^	JQ318804	(TC)10	F:<FAM > <Tail-3 > TCCGCTCTCCTCTCTGTCTC	138-164	11	0.8000	0.8441	0.8082	0.0003
			R:GTGAGTTGTGCTGCTGCAAT						
ZJU110^ab^	JQ318805	(AG)9	F:<FAM > <Tail-3 > TTGCACGGTGGTAGCTGTAG	143-159	5	0.7667	0.6486	0.5844	0.0000
			R:ACTGTGGTCCGTCGAACTCT						
ZJU111^ab^	JQ318806	(TC)8	F:<FAM > <Tail-3 > TTTCTAATGTTGTTCGCCCA	122-136	5	0.9000	0.5701	0.4652	0.0000
			R:TCATTCTCCTTGCAGATCCC						
ZJU112^ac^	JQ318807	(GA)8	F:<FAM > <Tail-3 > GGAGAGTGAGAGATCGCAGC	133-147	8	0.4839	0.6557	0.6212	0.0009
			R:GGCAACACCCTCAGTATCGT						
ZJU113^ab^	JQ318808	(AG)9	F:<FAM > <Tail-3 > AAACGCACCAGAGAAAGACG	138-154	6	0.6667	0.6588	0.5987	0.0130
			R:TCCATCTCTGGTCTCCATCC						
ZJU114^a^	JQ318809	(GA)10	F:<FAM > <Tail-3 > CTAGAGCGCTCCACGATACC	132-160	12	0.8214	0.8740	0.8448	0.0388
			R:AGAACGCTTGGAGAATCGAA						
ZJU115^ab^	JQ318810	(AG)14	F:<FAM > <Tail-3 > GGTCTGAGGCCTTCACTCTG	126-156	14	0.9677	0.9022	0.8775	0.0068
			R:GAGACCCAATAACCCATCCA						
ZJU116^ab^	JQ318811	(AG)15	F:<FAM > <Tail-3 > CTTTCTCCGTCTGCTCCATC	110-136	13	0.6875	0.8199	0.7846	0.0001
			R:GTCCAAACTTGGAGCCCATA						
ZJU117^ab^	JQ318812	(AAG)9	F:<FAM > <Tail-3 > TCTCAGATCCCTCCACGTTC	118-133	6	0.4688	0.6944	0.6426	0.0000
			R:CCACTGGATCAGGACAACCT						
ZJU118^ab^	JQ318813	(CT)9	F:<FAM > <Tail-3 > CAAGCCACGTGCATACCTATT	120-144	11	0.8750	0.8502	0.8171	0.0001
			R:CAGCTGGCTTCTAACTGCAA						
ZJU119^a^	JQ318814	(AG)11	F:<FAM > <Tail-3 > CTTTCGACTTCAGAGGCAGC	136-152	9	0.4828	0.8348	0.7975	0.0000
			R:TCCCTCTCAAACTTTGCCAC						
ZJU120^ab^	JQ318815	(GA)8	F:<HEX > <Tail-4 > TTGGTTTCGTTTGCAAGTCA	164-180	6	0.9355	0.7012	0.6354	0.0073
			R:GTCATCCATCCAATCCATCC						
ZJU121^a^	JQ318816	(CT)11	F:<HEX > <Tail-4 > AATCACCGAAGAAATCCACG	164-186	11	0.8621	0.8705	0.8426	0.0000
			R:ATTGCCCTCCCTTCTGTTCT						
ZJU122^ab^	JQ318817	(TC)8	F:<HEX > <Tail-4 > TGACGGAAGGATACTGGCTC	164-180	7	0.7742	0.7509	0.7012	0.0000
			R:CCATCAGACATGGCTTTCCT						
ZJU123^ab^	JQ318818	(CT)8	F:<HEX > <Tail-4 > TGAATTATTCGGTTCCCTGG	172-176	3	0.4667	0.6367	0.5499	0.2152
			R:TGCTTCAGTTCCAAACGAAA						
ZJU124^ab^	JQ318819	(CT)10	F:<HEX > <Tail-4 > GTGGCAGCCTCTCTATCGTC	161-187	12	0.9355	0.8778	0.8498	0.0001
			R:ATGACGTACTGCCCTTGCTT						
ZJU125^ab^	JQ318820	(TC)8	F:<HEX > <Tail-4 > TAAGGGCAGTCAGACCAACC	164-186	4	0.2188	0.3884	0.3453	0.0000
			R:CTGCAGCCTACAATGATCCA						
ZJU126^ab^	JQ318821	(GA)10	F:<HEX > <Tail-4 > CCAATGTGGACAGGTGTGAG	173-193	11	0.9677	0.8535	0.8228	0.0000
			R:GGCAGTAGTCGCTTCCCATA						
ZJU127	JQ318822	(GC)10	F:<HEX > <Tail-4 > AGGATCCTTGTCACCACCAG	165-189	11	0.9259	0.8288	0.7900	0.0079
			R:AATTCTTCCTTCCCAGCCTC						
ZJU128^ab^	JQ318823	(AG)14	F:<HEX > <Tail-4 > CCCAATTGACACAAATTCCC	145-161	5	0.4194	0.5019	0.4496	0.1981
			R:TTGGCATAGCATTGTTCGTC						
ZJU129^ab^	JQ318824	(CT)10	F:<HEX > <Tail-4 > GAGGTGCAATTACGTGGCTT	161-189	10	0.7500	0.8031	0.7611	0.0234
			R:TCAAGCATCAGCTGCTCAGT						
ZJU130^ab^	JQ318825	(GA)8	F:<HEX > <Tail-4 > GATTGCATGCACCAAATCAC	160-176	5	0.3478	0.4599	0.4131	0.2852
			R:GAATGTCCACGACGTGAATG						
ZJU131^ab^	JQ318826	(CT)14	F:<HEX > <Tail-4 > TTGAGAATCACAAACGCCTG	153-187	13	0.8710	0.8990	0.8735	0.0009
			R:GGTGGGTGAAATGCCTAGAA						
ZJU132^ab^	JQ318827	(CT)11	F:<HEX > <Tail-4 > AGGCACCTTTCTTTCCTCTC	164-178	5	0.6452	0.6568	0.5834	0.6586
			R:CAAGGAAGGAGGTGACGAAG						
ZJU133^ab^	JQ318828	(TC)11	F:<HEX > <Tail-4 > GCCCTGCAGTCTTTGTCAAT	171-195	8	0.8710	0.7731	0.7267	0.0000
			R:CAGCTTGCAGTGTTCATTCA						
ZJU134^ab^	JQ318829	(GA)11	F:<HEX > <Tail-4 > AGTGCCCAAGCATGACTTCT	172-190	8	0.9688	0.7907	0.7507	0.0004
			R:AATCAGTTGTCCGAGGATGG						
ZJU135^ab^	JQ318830	(AG)10	F:<HEX > <Tail-4 > AATTTACGGCTGTCCGTGAG	173-191	10	0.9688	0.7966	0.7557	0.0000
			R:CCTTGGGCTTCATGAACATT						
ZJU136^ab^	JQ318831	(GA)10	F:<HEX > <Tail-4 > TCCCACAGATCTCTAGCCGT	173-201	13	0.7742	0.8953	0.8692	0.0004
			R:CGCTCAGTTCTTAATTTCTTACTGTC						
ZJU137^ab^	JQ318832	(TC)8	F:<HEX > <Tail-4 > TGGATCTTGCTGCAGTTGTC	140-168	12	0.1875	0.6930	0.6612	0.0000
			R:AGCTAGCACTGGCCTAACCA						
ZJU138^ab^	JQ318833	(CT)10	F:<HEX > <Tail-4 > GCACAGTTGAGTTATGGGCA	152-170	8	0.3333	0.7746	0.7261	0.0001
			R:CTCTTTCAAATCCACGCACA						
ZJU139^ab^	JQ318834	(GA)12	F:<HEX > <Tail-4 > CCGAGCTTCGTTAGGACTTG	138-164	6	0.3667	0.4418	0.4043	0.0000
			R:CCAACAATACCCGAACCATC						
ZJU140^b^	JQ318835	(CT)14	F:<HEX > <Tail-4 > TGTGCTCATCTTGGATCCTG	172-198	9	0.6538	0.6139	0.5474	0.0000
			R:ACATCAGCTTGCATCCCTCT						
ZJU141^ab^	JQ318836	(CT)13	F:<HEX > <Tail-4 > CACAATCAGCTGCAGAATCAA	175-201	11	0.6774	0.7996	0.7600	0.0002
			R:AATGGCCGCTTGCAATATAA						
ZJU142^ab^	JQ318837	(TC)13	F:<HEX > <Tail-4 > CATTCACCTCCTTTCGCAAT	166-184	9	0.6774	0.6912	0.6498	0.0231
			R:ATCCAACGGCTCAAAGAATG						
ZJU143^ab^	JQ318838	(CT)12	F:<HEX > <Tail-4 > GTAGAGTAGATGCGCCTCGG	181-197	7	0.6923	0.7044	0.6397	0.0000
			R:ACGTACGAGCCATACACACG						
ZJU144^ab^	JQ318839	(AG)12	F:<HEX > <Tail-4 > GCCACTCTTCCCTCAACGTA	148-164	7	0.5161	0.6864	0.6252	0.0430
			R:CAGGTCAGTCCTGATGGGAT						
ZJU145^ab^	JQ318840	(CT)10	F:<HEX > <Tail-4 > TGTGGCTGTGTTCCTCCATA	155-175	7	0.6875	0.7351	0.6912	0.0000
			R:CAATGTTGGGTGCTTTGTTG						
ZJU146^ab^	JQ318841	(AG)10	F:<HEX > <Tail-4 > TGGAAACTTTGTCGTGTGGA	154-168	6	0.2258	0.6663	0.6187	0.0000
			R:TTATATCGGGCAGCCAGAAC						
ZJU147^ab^	JQ318842	(AG)10	F:<HEX > <Tail-4 > TTAGGAACCAAACTGGACGG	173-195	10	0.8333	0.7169	0.6811	0.0005
			R:TCAAATGCCGTGCTATTGAG						
ZJU148^ab^	JQ318843	(AG)18	F:<HEX > <Tail-4 > AAGAGCAGGAACCGAACCTT	160-190	15	0.9375	0.9067	0.8829	0.4973
			R:ACCGAAAGACGAAGAAACGA						
ZJU149^ab^	JQ318844	(TC)8	F:<HEX > <Tail-4 > AGCCCTCCATGTGTGCTTAT	139-163	11	0.8333	0.8718	0.8417	0.0022
			R:AGGGAGAGAGTGGTTCTGCC						
ZJU150^ab^	JQ318845	(AG)10	F:<HEX > <Tail-4 > ACTTAACTGAGAGGCTGCGG	163-201	10	0.9000	0.8469	0.8123	0.0053
			R:GTGGAAACCGAACGTCCTAA						
ZJU151^ab^	JQ318846	(CA)9	F:<HEX > <Tail-4 > GAATTGGAAATCCCTAGCCC	156-170	6	0.3750	0.5511	0.5188	0.0001
			R:CATTTGCGCATGTCTCCTTA						
ZJU152^ab^	JQ318847	(AG)11	F:<HEX > <Tail-4 > AAACGAAGTCGTTCAATGCC	163-181	7	0.9355	0.7578	0.7040	0.0161
			R:CTTGATTTGGGCCTTCGATA						
ZJU153^ab^	JQ318848	(AG)10	F:<HEX > <Tail-4 > CCAGCTCCGAATTAGCAAAC	173-191	6	1.0000	0.6667	0.5927	0.0000
			R:GTGGCGGTTTATCTCATCGT						
ZJU154^ab^	JQ318849	(AG)11	F:<HEX > <Tail-4 > TTGTCAATTGCCCTTCCTTC	156-184	10	0.9333	0.6847	0.6184	0.0000
			R:TTCCTCCCTTTCCCACTTCT						
ZJU155^ab^	JQ318850	(TC)9	F:<HEX > <Tail-4 > GAGAGCAATCAGTGAAGCCC	160-188	8	0.8438	0.6731	0.6037	0.0000
			R:GGGAGACGGATGTCGATTTA						
ZJU156^ab^	JQ318851	(TA)8	F:<HEX > <Tail-4 > ATACGTCGAAAGATCCACCG	166-184	7	0.5484	0.6626	0.6063	0.0000
			R:TTCTGGAATCCTTCCCATTG						
ZJU157^ab^	JQ318852	(AG)9	F:<HEX > <Tail-4 > CACTCACAACCAAAGCCAGA	154-186	13	0.9677	0.9064	0.8823	0.0171
			R:GTGCATAATCACAGGCATGA						
ZJU158^ab^	JQ318853	(AT)10	F:<HEX > <Tail-4 > CCAGATGATCACGCAGCTTA	156-174	9	0.6452	0.8292	0.7917	0.0000
			R:CGTCCTCCAATACGTTCCTC						
Mean					8.25	0.5636	0.7178	0.6730	

Note: ^a b c^ These SSRs are transferable for *M. adenophora, M. nana* and *M. cerifera*, respectively. SSR markers are listed according to ascending order in different fluorescent dyes. Shown for each primer pair are the repeat motif, primer sequences, size range (bp), number of alleles detected (*Na*), observed heterozygosity (*Ho*), expected heterozygosity (*He*), polymorphism information content (PIC) and Chi-square test for Hardy-Weinberg equilibrium (*P*_HW_). The annealing temperature was 60 **°**C; a, including length of tail sequences (18 bp total). *P*_HW_ over 0.05 are underlined.

The PIC at each locus ranged from 0.256 to 0.883 with an average of 0.67 loci. The PCR product size ranged from 110 to 274 bp. All the primers produced amplicons in agreement with the expected sizes. Most of the SSR primers (139 primer pairs) showed significant deviation from HW equilibrium (P < 0.05). Partial correlation analysis showed that significant positive correlations existed between the repeat unit length and PIC (P < 0.01, r = 0.2747). This showed that these SSRs had high rates of transferability for *M. adenophora* (91.14%) and *M. nana* (89.87%) and low rates for *M. cerifera* (46.84%), indicating that these markers are suitable for genetic diversity analyses in other *Myrica* species.

One of the objectives of this study was to develop potential suitable SSR markers for genetic mapping using Biqi and Dongkui as crossing parents. We selected 99 heterozygous loci in Biqi and 105 in Dongkui (Table 3): 135 primer pairs can be used together in linkage mapping of the planned F_1_ populations between Biqi and Dongkui.

**Table 3:** Distribution of the segregation types expected for the mapping population (Biqi × Dongkui)

**Segregation type**	**Alleles**	**Number**	**Mapping in F**_**1**_
aa × aa	1	12	No
aa × bb	2	11	No
aa × ab	2	24	Yes
ab × aa	2	18	Yes
ab × ab	2	8	Yes
aa × bc	3	12	Yes
ab × cc	3	12	Yes
ab × ac	3	41	Yes
ab × cd	4	20	Yes
Total		135	

### **Genetic relationship analysis**

**Figure 4:**
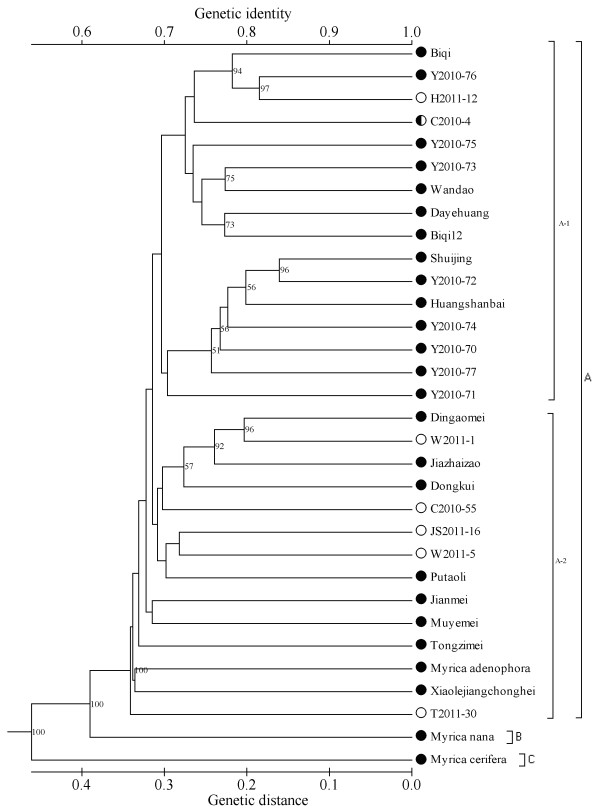
**Dendrogram for 32 Chinese bayberry accessions derived from UPGMA cluster analysis based on 158 SSR markers.** The symbols before the accession codes indicate the sex: ○, androphyte plant, ●, common cultivars, and ◘, monoecious plant. The numbers are bootstrap values based on 1000 iterations. Only bootstrap values larger than 50 are indicated.

The 32 accessions were divided into three groups (A, B and C, Figure 4), based on Nei’s genetic distance coefficient [16] using UPGMA cluster analysis. The similarity among all the accessions varied from 0.54 to 0.84. At the species level, the UPGMA dendrogram produced clusters separating *M. nana* and *M. cerifera* into two distinct groups. The genetic similarity between *M. cerifera* and *M. rubra* was 0.54, lower than the 0.74 previously reported by Xie [6]. 

The main cluster ‘A’ included the subgroups A-1 and A-2. Subgroup A-1 includes 16 accessions, mainly from the cities of Ningbo (12) and Hangzhou (3), where the popular and dominant cultivar is Biqi. This demonstrates that these natural elite seedling selections are truly distinct from the local cultivars. For their genetic relationships (Figure 4), the rare monoecious individual (C2010-4) is closely related to Biqi, while the accessions ‘Shuijing’ and ‘Y2010-72’ (both white fruit type) are clearly separated in the cluster, with low genetic distance.

Subgroup A-2 includes 14 accessions, with four from Wenzhou, two from Taizhou, and one each from the cities of Hangzhou and Ningbo, and the Hunan, Guangxi, Guizhou and Jiangsu provinces. This group includes the popular cultivar Dongkui. The four accessions from Wenzhou distributed in this cluster have genetic similarity. The accession ‘Tongzimei’ from the Hunan province is on an independent branch, showing that it is genetically distinct. ‘Xiaolejiangchonghei’ and ‘*M. adenophora*’ grouped together, and are possibly in the same population. Six androphyte accessions, distributed in group A, are close to cultivars of the same geographic origin.

The accessions ‘*Myrica nana*’ from Yunnan and ‘*Myrica cerifera*’ from the USA were independently classified as the ‘B’ and ‘C’ group, indicating a distant relationship with cultivated *Myrica rubra*.

## **Discussion**

Our major aims were to find a large set of SSR markers for *Myrica rubra* and understand the genetic diversity and relationship among representative cultivars, androphyate and related species. This research paves the way for constructing an SSR-based linkage map in *Myrica*.

### **The genome characteristics of genus*Myrica***

Shotgun sequencing is suitable for simple genomes, with no or few repeat sequences, such as Chinese bayberry. For such genomes, the genome can largely be assembled simply by merging together reads containing overlapping sequence [17]. We report the genome survey of Chinese bayberry using whole genome shotgun sequencing. The 17-nucleotide depth distribution suggests a genome size of 323 Mb, larger than peach (220 Mb, http://www.rosaceae.org/peach/genome), but close to our estimate of 250 Mb from flow cytometry using rice as the reference (date not shown). Although the highly homozygous material was selected on a limited number of SSR loci assays, the actual heterozygous rate, as revealed by 185 new SSR markers, was very high (63%). To overcome the key issue of heterozygosity and allow us to generate a high-quality genome sequence, we can use a unique homozygous form such as monoploid, derived using tissue culture or from nature and worth further study.

### **Marker development for under-utilised fruit crops**

SSRs have been widely used for high-throughput genotyping and map construction as they have the advantage of high abundance, random distribution within the genome, high polymorphism information content and stable co-dominance [18-20]. They can be developed from either genomic or expressed sequence tag (EST) libraries. Although EST-SSRs are useful for genetic analysis, their disadvantages of relatively low polymorphism and high concentration in gene-rich regions of the genome may limit their usage, especially for the construction of linkage maps [21]. In this study, a total of 600 SSR primer pairs were designed from 28,602 SSR sites, and 581 (96.8%) primer pairs were effective. Due to the self-complementary nature to form dimers, AT/TA is not usually used to develop markers [12]. Our findings are in agreement with that published for peach, where the dinucleotide repeat motifs were also found to be the most common, and (CT)_n_ as the most common repeat unit [22].

In the present study, the mean value of PIC was higher than the previously reported 0.62 [7], but the consistent relationship was observed between SSR polymorphism and repeat unit length. There are some reports of a positive relationship between degree of polymorphism and repeat unit length [23,24]. However, those polymorphic SSRs that are homozygous in both parents cannot be mapped in F_1_ populations, although they are useful for mapping in F_2_ or backcross populations [25], while heterozygous SSRs can be used for mapping in F_1_ populations (Table 2). The estimated number of SSRs that can be mapped in the F_1_ populations between Biqi and Dongkui was about 85%.

Recently, based on mass sequence data of Chinese bayberry obtained by RNA-Seq, 41,239 UniGenes were identified and approximately 80% of the UniGenes (32,805) were annotated, which provides an excellent platform for future EST-SSR development and functional genomic research [26].

### **High efficient test methods**

Normally, a universal M13 primer is labelled with one of a number of fluorescent dyes. The tailed primer provides a complementary sequence to the fluorescent labelled M13 primer, leading to the amplification of fluorescent PCR products, and then the PCR products of different sizes and/or labelled with different fluorescent dyes are mixed and tested [27]. In this research, a multiplex PCR strategy was designed using the universal M13-tailed primer and three additional tail primers, designed arbitrarily, in presumed four-plex amplifications in single PCR, for a major reduction in cost and time. However, as only a few primer combinations were successful, most resulting in weak bands, we did the PCR individually and mixed the PCR products. Further optimisation of multiplex PCR is needed to evaluate its general applicability.

### **Evolution of*Myrica*species**

In this study, both cultivated species and wild species were analysed and their genetic diversity could easily be differentiated. *M. nana* and *M. cerifera* were clearly genetically distant to *M. rubra*. *M. nana*, also known as the dwarf or Yunnan arbutus, is indigenous to the Yunnan and Guizhou provinces, and has a plant height of < 2 m. The juvenile period of fruit tree can be shortened for breeding purposes. Studies on embryo culture *in vitro* of the F_1_ seeds of crosses between *M. rubra* and *M. nana*, [28], has shown good cross compatibility between *M. rubra* and *M. nana*, resulting in 70.5% normal seeds with intact embryo. *M. adenophora* and *M. nana* grow as wild trees, with the fruit of *M. adenophora* only suitable for medical purposes and not edible.

Our findings on the genetic similarity between *M. adenophora* and *M. rubra,* which are considered a progenitor-derivative species pair, are consistent with a previously published figure of 0.897 [29]. An earlier study observed little change in allelic diversity along the chronosequence and no evidence for heterosis, although there was a moderate change in genotypic diversity [30]. The markers developed in this study can be very useful in future population structure analysis.

## **Conclusions**

In summary, the genome size of *Myrica* genus is small, about 320 Mb. A large set of SSRs were developed from a genome survey of *Myrica rubra*. The results suggest that they have high rates of transferability, making them suitable for use in other *Myrica* species.

## **Materials and methods**

### **Plant materials and genome survey**

We selected an androphyte ‘C2010-55’ for the genome survey because it was the most homozygous (10 out of 14 SSRs) individual among 230 accessions. Two DNA libraries of 250 and 500 bp insert size were constructed and sequenced by Illumina Hi-Seq 2000.

Twenty-nine accessions of the cultivated species (*M. rubra*) and 3 related species (*M. adenophora*, *M. nana*, *M. cerifera*), collected from different provinces in China (Table 4), were used to evaluate the suitability of the SSRs for genetic distance analysis. Young leaves were collected and frozen in liquid nitrogen prior to genomic DNA extraction using CTAB methods [4]. DNA concentrations were measured spectrophotometrically at 260 nm, and the extracts electrophoresed on 1% agarose to confirm the quality. The purified DNAs were standardised at 40 ng/μl and stored at -40°C. 

**Table 4:** The 32 bayberry accessions included in this study

**No.**	**Accession**	**Fruit/Flower colour**^**a**^	**Fruit maturity date**	**Region**
1	Biqi	black	Late June	Cixi, Ningbo, Zhejiang
2	Dongkui	red	Early July	Taizhou, Zhejiang
3	Dayehuang	red	Mid-June	Hangzhou, Zhejiang
4	Dingaomei	black	Mid to late June	Wenzhou, Zhejiang
5	Huangshanbai	white	Early July	Hangzhou, Zhejiang
6	Jiazhaizao	black	Mid-June	Wenzhou, Zhejiang
7	Jianmei	red	Late June	Cixi, Ningbo, Zhejiang
8	Muyemei	black	Late June	Jinhua, Zhejiang
9	Putaoli	black	Mid June	Hangzhou, Zhejiang
10	Shuijing	white	Late June/Early July	Yuyao, Ningbo, Zhejiang
11	Tongzimei	black	Mid-June	Hunan
12	Wandao	black	Early July	Zhoushan, Zhejiang
13	Xiaolejiangchonghei	black	May	Guizhou
14	Biqi12	black	Late June	Yuyao, Ningbo, Zhejiang
15	Y2010-70	red	Late June/Early July	Yuyao, Ningbo, Zhejiang
16	Y2010-71	black	Mid to late June	Yuyao, Ningbo, Zhejiang
17	Y2010-72	white	Late June/Early July	Yuyao, Ningbo, Zhejiang
18	Y2010-73	red	Late June	Yuyao, Ningbo, Zhejiang
19	Y2010-74	red	Late June/Early July	Yuyao, Ningbo, Zhejiang
20	Y2010-75	black	Late June	Yuyao, Ningbo, Zhejiang
21	Y2010-76	white	Late June/Early July	Yuyao, Ningbo, Zhejiang
22	Y2010-77	red	Late June/Early July	Yuyao, Ningbo, Zhejiang
23	C2010-4	red	Late June	Cixi, Ningbo, Zhejiang
24	*C2010-55	red	-	Cixi, Ningbo, Zhejiang
25	*W2011-1	yellow- red	-	Wenzhou, Zhejiang
26	*W2011-5	red	-	Wenzhou, Zhejiang
27	*H2011-12	yellow-green	-	Hangzhou, Zhejiang
28	*JS2011-16	red	-	Suzhou, Jiangsu
29	*T2011-30	red	-	Taizhou, Zhejiang
30	*Myrica adenophora*	red	February to May	Guilin, Guangxi
31	*Myrica nana*	red	June to July	Yunnan
32	*Myrica cerifera	yellow-green	-	Cixi, Ningbo, Zhejiang

Note: fruit colour for cultivar and flower colour for androphyte. * selected androphytes.

### **SSR identification and primer design**

We used MISA scripting language ( http://pgrc.ipk-gatersleben.de/misa/misa.html) to identify microsatellite repeats in our sequence database. The SSR loci containing perfect repeat units of 2-6 nucleotides only were considered. The minimum SSR length criteria were defined as six reiterations for dinucleotide, and five reiterations for other repeat units. Mononucleotide repeats and complex SSR types were excluded from the study.

The SSR primers were designed using BatchPrimer3 interface modules ( http://pgrc.ipk-gatersleben.de/misa/primer3.html). We selected 600 primers that met the following parameters: 110–230 final product length, primer size from 18 to 22 bp with an optimum size of 20 bp, and the annealing temperature was set at 60°C. The repeat units over eight were used.

Tail-1(M13 universal sequence-TGTAAAACGACGGCCAGT), Tail-2(CGAGTCAGTATAGGGCAC), Tail-3(ATCACGAAGCAGATGCAA) and Tail-4(GAGCATCTCGTACCAGTC) were added to the 5’ end of each 150 forward primers of pairs respectively. Tail-2, Tail-3 and Tail-4 were designed so that the primer size was 18 bp and the annealing temperature was 53°C. Primers were synthesised by Invitrogen Trading (Shanghai) Co., Ltd. We primarily tested two cultivars (Biqi and Dongkui) and *M. cerifera* for 600 SSR loci by PAGE (polyacrylamide denaturing gel) to confirm their suitability. Tail-1, Tail-2, Tail-3 and Tail-4 labelled with one of the following dyes: NED, PET, FAM, and HEX, respectively.

### **Polymerase chain reaction and gel electrophoresis**

Each 20 μl reaction mixture contained 10 × PCR buffer (plus Mg2+), 0.2 mM of each dNTP, 5 pmol of each reverse, 4 pmol of the tail primer, 1 pmol of the forward primer, 0.5 units of rTaq polymerase (TaKaRa, China) and 40 ng genomic DNA template. Each primer pair had an interval of 20 bp according to the expected size of amplicons.

DNA amplification was in an Eppendorf Mastercycler (Germany) programmed at 94°C for 5 min for initial denaturation, then 32 cycles at 94°C (30 s)/58°C (30 s)/72°C (30 s), followed by 8 cycles of 94°C (30 s)/53°C (30 s)/72°C (30 s). The final extension step was 10 min at 72°C. Each PCR product was run on 1% agarose gel at 110 V for a quality check.

Subsequently, PCR products were electrophoresed on 8% denaturing PAGE, according to Myers et al. [31], at 60 W in a Sequi-Gen GT Nucleic Acid electrophoresis cell (BioRad) for 4 h, depending on the fragment sizes to be separated, and visualised by silver staining [32]. Genotypes showing one and two bands were scored as homozygous and heterozygous, respectively, and the results recorded and photographed.

Multiplex PCR was designed and tested with products of different sizes and labelled with different fluorescent dyes. Each 20 μl reaction mixture contained 10 × PCR buffer (plus Mg^2+^), 0.8 mM of each dNTP, 1 unit of rTaq polymerase, 40 ng genomic DNA template and a total of four primer pairs with 5 pmol of each reverse primer, 4 pmol of each tail primer, and 1 pmol of each forward primer. The PCR products were diluted, mixed with the internal size standard LIZ500 (Applied Biosystems) and loaded on an ABI 3130 Genetic Analyzer. Alleles were scored using GeneMapper version 4.0 software (Applied Biosystems, Foster City, CA, USA).

### **Data analysis**

The raw genome sequence data was first filtered to obtain high quality reads, then assembled using SOAP ( http://soap.genomics.org.cn/soapdenovo.html) denovo software to contig, scaffold and fill in gaps. In addition, we used SSPACE software to build the scaffold. K-mer analysis was to help estimate the genome size and characters, such as heterozygosis and repeats.

The number of alleles (A), observed heterozygosity (Ho) and expected heterozygosity (He) were calculated using POPGENE version 1.32 ( http://www.ualberta.ca/~fyeh/popgene_download.html). Chi-square test for Hardy-Weinberg equilibrium allele frequencies and polymorphism information content (PIC) was calculated using PowerMarker version 3.25 [33] ( http://statgen.ncsu.edu/powermarker/downloads.htm). Microsoft office Excel 2007 was used to analyse the correlation. Genetic similarity among all the accessions was calculated according to Dice’s coefficients using Nei's coefficient index [16] with the Free Tree 0.9.1.50 [34] ( http://www.natur.cuni.cz/~flegr/programs/freetree.htm) software, and the dendrogram constructed using the unweighted pair-group method with arithmetic averaging (UPGMA) option. The confidence of branch support was then evaluated by bootstrap analysis with 1,000 replicates. The dendrogram was printed using MEGA version 5.05 software [35] ( http://www.megasoftware.net/mega.php).

## Authors’ contributions

ZSG: HJJ: EVW and MLC designed the experiments. YJ: CYC: GYW collected plant materials. YJ: HMJ and XWL performed the SSR experiments and analysed the data. The whole genome shotgun and sequencing assembly was performed by ZC. YJ: ZSG and EVW drafted this manuscript.

## Supplementary Material

Additional file 1 Occurrence of different SSRs in the genome survey of Chinese bayberry. Click here for file

## Author details

^1^Department of Horticulture, The State Agriculture Ministry Laboratory of Horticultural Plant Growth, Development and Quality Improvement, Zhejiang University, Hangzhou 310058, China. ^2^BGI-Shenzhen, Beishan Industrial Zone, Yantian District, Shenzhen 518083, China. ^3^Fruit Research Institute, Yuyao, Ningbo 315400, China. ^4^Forestry Technology Extension Center, Cixi Ningbo 315300, China. ^5^Plant Breeding-Wageningen University and Research Centre, P.O. Box 166700 AA, Wageningen, The Netherlands.
