# Supplementary material for: Development of simple sequence repeat (SSR) markers from a genome survey of Chinese bayberry (Myrica rubra)
Source: BMC Genomics. 2012 May 23;13:201. doi: 10.1186/1471-2164-13-201 (PMC3505174; doi:10.1186/1471-2164-13-201)
Supplement: Additional file 1 — Occurrence of different SSRs in the genome survey of Chinese bayberry. [file 1471-2164-13-201-S1.doc]

**Additional file 1** Occurrence of different SSRs in Genome Survey to Chinese bayberry

| SSR Motif | Number of repeats | | | | | | | | | | | | Total |
| --- | --- | --- | --- | --- | --- | --- | --- | --- | --- | --- | --- | --- | --- |
|  | 5 | 6 | 7 | 8 | 9 | 10 | 11 | 12 | 13 | 14 | 15 | ≧16 |  |
| AC/GT | - | 597 | 453 | 297 | 150 | 75 | 23 | 11 | 14 | 5 | 3 | 18 | 1646 |
| AG/CT | - | 4245 | 3981 | 2042 | 1138 | 787 | 314 | 132 | 87 | 79 | 76 | 137 | 13018 |
| AT/AT | - | 3798 | 3077 | 1305 | 667 | 365 | 132 | 53 | 27 | 19 | 13 | 43 | 9499 |
| CG/CG | - | 43 | 15 | 7 | 1 | 3 | 0 | 0 | 0 | 0 | 0 | 1 | 70 |
| AAC/GTT | 145 | 55 | 13 | 0 | 0 | 0 | 0 | 0 | 0 | 0 | 0 | 0 | 213 |
| AAG/CTT | 998 | 327 | 114 | 10 | 8 | 2 | 0 | 2 | 1 | 0 | 1 | 1 | 1464 |
| AAT/ATT | 850 | 304 | 118 | 4 | 4 | 1 | 0 | 0 | 0 | 0 | 0 | 2 | 1283 |
| ACC/GGT | 125 | 55 | 13 | 1 | 0 | 0 | 0 | 0 | 0 | 0 | 0 | 0 | 194 |
| ACG/CGT | 51 | 12 | 6 | 0 | 0 | 0 | 0 | 0 | 0 | 0 | 0 | 0 | 69 |
| ACT/AGT | 26 | 13 | 1 | 0 | 0 | 0 | 0 | 0 | 0 | 0 | 0 | 0 | 40 |
| AGC/CTG | 116 | 27 | 3 | 0 | 0 | 0 | 0 | 0 | 0 | 0 | 0 | 0 | 146 |
| AGG/CCT | 195 | 75 | 26 | 3 | 0 | 0 | 0 | 0 | 0 | 0 | 0 | 0 | 299 |
| ATC/ATG | 146 | 51 | 5 | 0 | 0 | 0 | 0 | 0 | 0 | 1 | 0 | 0 | 203 |
| CCG/CGG | 26 | 3 | 1 | 0 | 0 | 0 | 0 | 0 | 0 | 0 | 0 | 0 | 30 |
| AAAC/GTTT | 27 | 4 | 0 | 0 | 0 | 0 | 1 | 0 | 0 | 0 | 0 | 0 | 32 |
| AAAG/CTTT | 73 | 6 | 0 | 0 | 0 | 0 | 0 | 0 | 0 | 0 | 0 | 0 | 79 |
| AAAT/ATTT | 94 | 11 | 0 | 0 | 0 | 0 | 0 | 1 | 1 | 0 | 0 | 0 | 107 |
| AACC/GGTT | 8 | 0 | 0 | 0 | 0 | 0 | 0 | 0 | 0 | 0 | 0 | 0 | 8 |
| AACG/CGTT | 1 | 0 | 1 | 0 | 0 | 0 | 0 | 0 | 0 | 0 | 0 | 0 | 2 |
| AAGC/CTTG | 1 | 0 | 0 | 0 | 0 | 0 | 0 | 0 | 0 | 0 | 0 | 0 | 1 |
| AAGG/CCTT | 11 | 1 | 0 | 0 | 0 | 0 | 0 | 0 | 0 | 0 | 0 | 0 | 12 |
| AATC/ATTG | 7 | 1 | 0 | 0 | 0 | 0 | 0 | 0 | 0 | 0 | 0 | 0 | 8 |
| AATG/ATTC | 4 | 0 | 0 | 0 | 0 | 0 | 0 | 0 | 0 | 0 | 0 | 0 | 4 |
| AATT/AATT | 17 | 0 | 1 | 0 | 0 | 0 | 0 | 0 | 0 | 0 | 0 | 0 | 18 |
| ACAG/CTGT | 7 | 1 | 0 | 1 | 0 | 0 | 0 | 0 | 0 | 0 | 0 | 0 | 9 |
| ACAT/ATGT | 8 | 3 | 0 | 0 | 0 | 0 | 0 | 0 | 0 | 0 | 0 | 0 | 11 |
| ACCC/GGGT | 11 | 0 | 0 | 0 | 0 | 0 | 0 | 0 | 0 | 0 | 0 | 0 | 11 |
| ACCG/CGGT | 2 | 1 | 0 | 0 | 0 | 0 | 0 | 0 | 0 | 0 | 0 | 0 | 3 |
| ACCT/AGGT | 1 | 0 | 0 | 0 | 0 | 0 | 0 | 0 | 0 | 0 | 0 | 0 | 1 |
| ACGC/CGTG | 3 | 0 | 0 | 0 | 0 | 0 | 0 | 0 | 0 | 0 | 0 | 0 | 3 |
| ACGT/ACGT | 2 | 0 | 0 | 0 | 0 | 0 | 0 | 0 | 0 | 0 | 0 | 0 | 2 |
| ACTC/AGTG | 2 | 1 | 0 | 0 | 0 | 0 | 0 | 0 | 0 | 0 | 0 | 0 | 3 |
| ACTG/AGTC | 3 | 1 | 0 | 0 | 0 | 0 | 0 | 0 | 0 | 0 | 0 | 0 | 4 |
| AGAT/ATCT | 10 | 3 | 1 | 0 | 0 | 0 | 0 | 0 | 0 | 0 | 0 | 1 | 15 |
| AGCC/CTGG | 3 | 0 | 0 | 0 | 0 | 0 | 0 | 0 | 0 | 0 | 0 | 0 | 3 |
| AGCG/CGCT | 2 | 1 | 0 | 0 | 0 | 0 | 0 | 0 | 0 | 0 | 0 | 0 | 3 |
| AGCT/AGCT | 8 | 1 | 0 | 0 | 0 | 0 | 0 | 0 | 0 | 0 | 0 | 0 | 9 |
| AGGC/CCTG | 2 | 0 | 0 | 0 | 0 | 0 | 0 | 0 | 0 | 0 | 0 | 0 | 2 |
| AGGG/CCCT | 16 | 6 | 0 | 0 | 0 | 0 | 0 | 0 | 0 | 0 | 0 | 0 | 22 |
| ATCC/ATGG | 4 | 1 | 0 | 0 | 0 | 0 | 0 | 0 | 0 | 0 | 0 | 0 | 5 |
| ATCG/ATCG | 1 | 0 | 0 | 0 | 0 | 0 | 0 | 0 | 0 | 0 | 0 | 0 | 1 |
| ATGC/ATGC | 5 | 0 | 0 | 0 | 0 | 0 | 0 | 0 | 0 | 0 | 0 | 0 | 5 |
| AAAAC/GTTTT | 2 | 0 | 0 | 0 | 0 | 0 | 0 | 0 | 0 | 0 | 0 | 0 | 2 |
| AAAAG/CTTTT | 9 | 0 | 0 | 0 | 0 | 0 | 0 | 0 | 0 | 0 | 0 | 0 | 9 |
| AAAAT/ATTTT | 6 | 0 | 0 | 0 | 0 | 0 | 0 | 0 | 0 | 0 | 0 | 0 | 6 |
| AAACC/GGTTT | 1 | 0 | 0 | 0 | 0 | 0 | 0 | 0 | 0 | 0 | 0 | 0 | 1 |
| AAATC/ATTTG | 1 | 0 | 0 | 0 | 0 | 0 | 0 | 0 | 0 | 0 | 0 | 0 | 1 |
| AAATT/AATTT | 1 | 0 | 0 | 0 | 0 | 0 | 0 | 0 | 0 | 0 | 0 | 0 | 1 |
| AACAC/GTGTT | 1 | 0 | 0 | 0 | 0 | 0 | 0 | 0 | 0 | 0 | 0 | 0 | 1 |
| AAGAC/CTTGT | 1 | 0 | 0 | 0 | 0 | 0 | 0 | 0 | 0 | 0 | 0 | 0 | 1 |
| AAGAG/CTCTT | 1 | 0 | 0 | 0 | 0 | 0 | 0 | 0 | 0 | 0 | 0 | 0 | 1 |
| AAGCC/CTTGG | 1 | 0 | 0 | 0 | 0 | 0 | 0 | 0 | 0 | 0 | 0 | 0 | 1 |
| AAGGG/CCCTT | 1 | 0 | 0 | 0 | 0 | 0 | 0 | 0 | 0 | 0 | 0 | 0 | 1 |
| AATCT/AGATT | 1 | 0 | 0 | 0 | 0 | 0 | 0 | 0 | 0 | 0 | 0 | 0 | 1 |
| AATTC/AATTG | 1 | 0 | 0 | 0 | 0 | 0 | 0 | 0 | 0 | 0 | 0 | 0 | 1 |
| ACCTC/AGGTG | 1 | 0 | 0 | 0 | 0 | 0 | 0 | 0 | 0 | 0 | 0 | 0 | 1 |
| ACTGG/AGTCC | 1 | 0 | 0 | 0 | 0 | 0 | 0 | 0 | 0 | 0 | 0 | 0 | 1 |
| AGAGG/CCTCT | 1 | 0 | 0 | 0 | 0 | 0 | 0 | 0 | 0 | 0 | 0 | 0 | 1 |
| AGATG/ATCTC | 1 | 0 | 0 | 0 | 0 | 0 | 0 | 0 | 0 | 0 | 0 | 0 | 1 |
| AGCCC/CTGGG | 3 | 0 | 0 | 0 | 0 | 0 | 0 | 0 | 0 | 0 | 0 | 0 | 3 |
| AGCCT/AGGCT | 1 | 0 | 0 | 0 | 0 | 0 | 0 | 0 | 0 | 0 | 0 | 0 | 1 |
| AAAAAC/GTTTTT | 0 | 1 | 0 | 0 | 0 | 0 | 0 | 0 | 0 | 0 | 0 | 0 | 1 |
| AAAAAG/CTTTTT | 2 | 0 | 0 | 0 | 0 | 1 | 0 | 0 | 0 | 0 | 0 | 0 | 3 |
| AAACCT/AGGTTT | 1 | 0 | 0 | 0 | 0 | 0 | 0 | 0 | 0 | 0 | 0 | 0 | 1 |
| AAAGGG/CCCTTT | 1 | 0 | 0 | 0 | 0 | 0 | 0 | 0 | 0 | 0 | 0 | 0 | 1 |
| ACCCCC/GGGGGT | 1 | 0 | 0 | 0 | 0 | 0 | 0 | 0 | 0 | 0 | 0 | 0 | 1 |
| AGAGGG/CCCTCT | 0 | 0 | 1 | 0 | 0 | 0 | 0 | 0 | 0 | 0 | 0 | 0 | 1 |
| AGCCCC/CTGGGG | 1 | 0 | 0 | 0 | 0 | 0 | 0 | 0 | 0 | 0 | 0 | 0 | 1 |
| AGCCTG/AGGCTC | 0 | 1 | 0 | 0 | 0 | 0 | 0 | 0 | 0 | 0 | 0 | 0 | 1 |
| Total | 3052 | 9649 | 7830 | 3670 | 1968 | 1234 | 470 | 199 | 130 | 104 | 93 | 203 | 28602 |
